# HPLC-DAD-ELSD Combined Pharmacodynamics and Serum Medicinal Chemistry for Quality Assessment of Huangqi Granule

**DOI:** 10.1371/journal.pone.0123176

**Published:** 2015-04-27

**Authors:** Huaguo Chen, Xin Zhou, Yang Zhao, Xiao-Jian Gong, Yan He, Feng-Wei Ma, Mei Zhou, Chao Zhao, Yi Niu, Jie Deng

**Affiliations:** 1 Guizhou Engineering Laboratory for Quality Control & Evaluation Technology of Medicine, Guiyang, Guizhou, P. R. China; 2 The Research Center for Quality Control of Natural Medicine, Guizhou Normal University, Guiyang, Guizhou, P. R. China; 3 Key laboratory for Information System of Mountainous Areas and Protection of Ecological Environment, Guizhou Normal University, Guiyang, Guizhou, P. R. China; 4 Han Fang Pharmaceutical co., LTD of Guizhou province, Guiyang, Guizhou, P. R. China; Cardiff University, UNITED KINGDOM

## Abstract

**Objective:**

To more scientifically and reasonably control the quality of Huangqi Granules, preliminary studies on the pharmacodynamics and serum pharmacochemistry of this medicine were performed. DPPH and MTT experiments showed that water extracts of Huangqi Granules had good antioxidant activity and increased immunity. Timed blood samples collected 5 min, 15 min, and 30 min after oral administration of a set amount of Huangqi Granules were collected and tested using UPLC-ESI-MS/MS. As a result, calycosin-7-O-*β*-D-glucoside, ononin, calycosin, astragaloside IV, and formononetin were found to exist in rat blood after dosing, indicating that the five chemical compounds might have pharmacological activity, and based on this result, they were designated biomarkers for quality control of Huangqi Granules. Consequently, a simple, rapid and efficient method was developed in the present study for the simultaneous determination of the five characteristic compounds in Huangqi Granules using *HPLC-DAD-ELSD*.

**Materials and Methods:**

The separation was performed using an Agilent Hypersil ODS column (4.6 × 250 mm, 5 *μ*m) at 30 ℃. The mobile phase was composed of water (solvent A) and acetonitrile (solvent B) with a flow rate of 1 mL/min. The drift tube temperature of the ELSD system was set to 85 ℃, and the nitrogen pressure was 3.5 bar.

**Results:**

All five characteristic compounds had good linear behavior with r2 values greater than 0.9972. The recoveries varied from 96.31% to 101.22%. Subsequently, the developed method was applied to evaluate the quality of Huangqi Granules from different batches, and hierarchical clustering analysis (HCA) was used to analyze the classification of the samples based on the values of the five compounds.

**Conclusion:**

The established HPLC method combined with HCA proved to be effective to evaluate the quality of Huangqi Granules.

## Introduction

Huangqi Granules, a commercially formulated product, contain only *Radix Astragali* extract and some auxiliary materials. *Radix Astragali* (RA, called Huangqi in Chinese), the dried root of *Astragalus membranaceus* (Fisch.) Bge. var. *mongholicus* (Bge.) Hsiao or *A*. *membranaceus* (Fisch.) Bge., has a long history of medicinal use within the traditional Chinese system and is officially listed in the Chinese Pharmacopoeia [1] for “Qi tonifying” or adaptogenic use [2, 3]. RA has demonstrated a wide range of pharmacological effects and has been proven to have efficacious biological functions, including antiviral [4], hepatoprotective, antitumor [5], diuretic, antidiabetic, antioxidant [6] and analgesic activities, and to act as an immunostimulant [3].

As researchers identify and isolate bioactive components, the understanding of RA’s physiological, therapeutic, and clinical actions increases [7]. The constituents most often associated with the activity of RA are attributable to isoflavonoids [8], triterpene saponins [9], polysaccharides [10] and various trace elements. Isoflavonoids are a group of beneficial components that originate from nature and have aromatic structures, making them UV chromophores and, therefore, amenable to common HPLC detectors [2]. In general, the major isoflavonoids in RA are formononetin, ononin, calycosin and its glycosides, which are considered “marker compounds”; they possess antioxidant properties and can be used to treat cardiovascular diseases [11]. Astragaloside IV, a major naturally occurring saponin isolated from RA, is used for the quality evaluation of RA in Chinese Pharmacopoia [1]. Recent studies have shown that Astragaloside IV has many pharmacologic uses, including cardioprotection during myocardial ischemia [12, 13], protection against ischemic brain injury [14], anti-inflammatory activity [15], and free-radical scavenging activity [16]. Unlike isoflavonoids, which have chromophore groups, the astragalosides in RA have poor absorption of UV radiation and are difficult to detect using this type of detector [17]. Because most analytes are less volatile than LC mobile phases, ELSD provides nearly universal detection [18, 19] and is, therefore, used as an alternative detector to determine these non-chromophoric compounds.

Many analytical methods have been adopted to evaluate the quality of RA, either through qualitative and quantitative analysis of isoflavonoids using HPLC-UV [20] and LC-MS [2] or through analysis of saponins using HPLC-ELSD and LC-MS [21] or both isoflavonoids and saponins through HPLC-UV-ELSD [17, 22–24] and LC-MS. As many as possible of the chemical constituents should be quantified for comprehensive quality control of a medical plant. However, as researchers in the field of TCM quality control, we should determine if the chemical compounds that were selected for quality control of an herb are exactly the ones absorbed into the patients’ blood and responsible for the pharmacological effects [25]. In this case, determining the absorbed chemical compounds is a prerequisite for subsequent quality control of RA or its commercially formulated products, and based on this point, a serum pharmacochemistry study on Huangqi Granules was performed to screen for the absorbed constituents in the present study. As a result, calycosin-7-O-β-D-glucoside, ononin, calycosin, astragaloside IV, and formononetin were found to exist in rats’ blood after dosing, indicating that the five chemical compounds might have pharmacological activity and were, therefore, designated biomarkers for quality control of Huangqi Granules.

**Fig 1:**
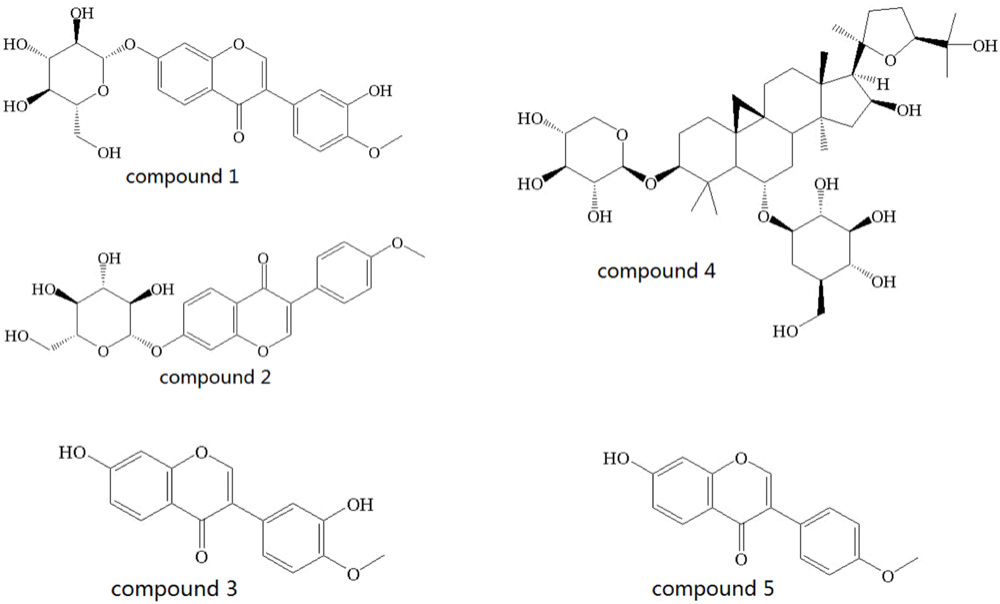
The structures of the five characteristic compounds. (1) Calycosin-7-O-*β*-D-glucoside; (2) Ononin; (3) Calycosin; (4) Astragaloside IV; and (5) Formononetin

In the present study, a quantitative analytical method that simultaneously measures calycosin-7-O-*β*-D-glucoside, ononin, calycosin, astragaloside IV, and formononetin (the structures are shown in Fig 1) using HPLC combined with both DAD and ELSD was established. This simple method proved to be efficient in the evaluation of the quality of Huangqi Granules.

## Experiment

### Chemicals and reagents

Authenticated reference compounds of calycosin-7-O-*β*-D- glucoside, ononin, calycosin, astragaloside IV, and formononetin were purchased from Chengdu Ruifensi Company (Chengdu, China). The LC-grade acetonitrile was from TEDIA Company (Fairfield, OH, USA). ROBUST purified water was used as the mobile phase. All other reagents were of analytical grade. Thirty batches of Huangqi Granule were supplied by the Guizhou Hanfang Pharmaceutical Company.

### Preparation of standard and sample solutions

The authenticated reference compounds of calycosin-7-O-β-D- glucoside, ononin, calycosin, astragaloside IV, and formononetin were accurately weighed and dissolved in methanol to prepare stock solutions (0.562, 0.1785, 0.254, 1.121 and 0.0274 mg/mL, respectively). The working solutions were obtained by diluting the stock solutions with methanol to a series of proper concentrations. All of the stock and working solutions were stored at 4°C for analysis.

First, 0.8 g of pulverized Huangqi Granules was accurately weighed and transferred into a 10-mL volumetric round bottom flask with 30% methanol (v/v), and the samples were then ultrasonicated at ambient temperature for 1 h (100 W, 40 kHz); 30% methanol was then added to compensate for the weight lost during the extraction. The extract was filtered through a 0.45-*μ*m microporous membrane filter for analysis. All of the sample solutions were stored at 4°C.

### Antioxidant capacity assay of Huangqi Granule

A DPPH assay was used to evaluate the antioxidant capacity of Huangqi Granules in this study. Different batches of Huangqi Granule samples were dissolved with distilled water at different concentrations. The DPPH free radical scavenging activity of each extract was determined according to the method described by Della with slight modification. Briefly, a 0.1 mM ethanol DPPH solution was prepared. The initial absorbance of the DPPH in ethanol was measured at 517 nm and did not change throughout the assay. An aliquot (0.1 ml) of each extract (with appropriate dilution if necessary) was mixed with 2.0 ml of the ethanol DPPH solution and incubated for 40 min at 25°C in the dark. Then, the absorbance at 517 nm was measured. The DPPH free radical scavenging activity is expressed using the IC_50_ value, which is the concentration of the sample required to decrease the absorbance at 517 nm by 50%. The measurements were performed in triplicate.

### MTT assay of Huangqi Granule

Murine macrophages (RAW 264.7 cells) were purchased from the Korean Cell Line Bank (Seoul, Korea). They were cultured in Dulbecco’s modified Eagle’s medium (DMEM) at 37°C in a humidified incubator with an atmosphere of 5% CO_2_. Cell viability was evaluated using the MTT (3-(4,5-dimethylthiazol-2-yl)-5-(3-carboxymethoxyphenyl-2- (4-sulfo-phenyl)-2H-tetrazolium) (Sigma—Aldrich, US) assay. After 2 h of adhesion of RAW 264.7 cells according to the experimental protocol, the different batches of the Huangqi Granule extracts were plated with a total volume of 200 μL in 96-well plates. The wells containing only RAW 264.7 cells were used as the control groups. After 24 h of incubation at 37°C, 20 μL of MTT (final concentration 5 mg/mL) was added to each well, and the plates were incubated for 4 h; then, 150 μL of DMSO was added to dissolve the blue formazan crystals. The absorbance was measured using spectrophotometry at 570 nm.

### Ethics Statement

This study was approved by the Animal Ethics Committee of Guizhou normal University.

### Serum pharmacochemistry study of Huangqi Granules

Six male Sprague Dawley (SD) rats weighing 250 ± 20 g were obtained from Lab Animal Ltd. (Changsha, China) and kept in air-conditioned animal quarters (temperature, 22 ± 2°C; humidity, 70 ± 10%). These SD rats were housed in a plastic walled cage with unlimited access to food and water except for 12 h before and during the experiments. The protocols for the animal experiments were reviewed and approved by the Guizhou normal University Animal Ethics Committee. Each rat was intragastrically administered the Huangqi Granule extract (0.38 g/ml) at a dose of 2 ml/100 g body weight.

Before administration and 5 min, 15 min, and 30 min after oral administration of the extract, a short piece of heparinized capillary tubing was used to puncture the orbital sinus at the posterior angle of the eye, and approximately 300-*μ*l blood samples were collected and immediately centrifuged at 3000 rpm for 10 min at room temperature. Then, 400 *μ*l of “methanol/acetonitrile” (1:3, v/v) was added to 100 *μ*l of the serum samples and stirred well. The mixture was centrifuged at 12000 rpm for 10 min at 4°C, and the supernatant was evaporated to dryness at 40°C under gentle streams of nitrogen. The dry residue was reconstituted in 80 *μ*l of high purity water (brief vortex) followed by 20 *μ*l of methanol, then centrifuged again at 12000 rpm for 10 min at 4°C, and 10 *μ*l of the supernatant was used for LC-MS/MS analysis.

### Instrumentation and chromatographic conditions

LC-MS/MS analyses of serum samples were performed on an Accela 1250 UHPLC system equipped with an Accela 1250 PDA detector, an Accela HTC Pal autosampler, and an Accela 1250 binary pump. The chromatographic separations were achieved on a Hypersil GOLD aQ-C18 column (2.1 × 50 mm, 1.9 *μ*m). The mobile phase consisted of a 0.1% aqueous formic solution (B) and 0.1% formic methanol (C) with the following gradient elution program: 0–4 min, 25% C; 4–12 min, 25–90% C; 12–12.1 min, 90–25% C; and 12.1–21 min, 25% C. The column temperature was maintained at 25°C with a flow rate of 200 *μ*L/min, and the injection volume was 10 *μ*L. The mass spectrometric analyses were performed on a TSQ quantum ultratriple-quadrupole mass spectrometer (Thermo Fisher Scientific Inc., Waltham, MA, USA) equipped with an electrospray ionization interface. The MS instrument parameters were as follows: sheath gas flow rate, 45 psi; auxiliary gas flow rate, 15 (arbitrary units); spray voltage, 3000 V; vaporizer temperature, 500°C; and capillary temperature, 350°C. Helium was used as the collision gas for CID, and the collision energy, tube lens offset, and collision pressure for each parent ion-product ion transition are displayed in Table 1.

**Table 1:** The collision energy, tube lens offset, and collision pressure for each parent ion-product ion transition.

Analytes	Transition	Tube Lens Offset (V)	Collision Pressure (m Torr)	Collision Energy (eV)
Formononetin	266.986→195.234	81	1.5	39
Formononetin	266.986→223.206	81	1.5	34
Formononetin	266.986→252.224	81	1.5	22
Calycosin	283.156→211.211	76	1.5	35
Calycosin	283.156→239.196	76	1.5	35
Calycosin	283.156→268.203	76	1.5	22
Ononin	430.890→253.985	79	1.5	42
Ononin	430.890→269.134	79	1.5	19
Calycosin-7-O-*β*-D-glucoside	447.097→269.993	90	1.5	37
Calycosin-7-O-*β*-D-glucoside	447.097→285.029	90	1.5	18
Astragaloside IV	807.159→203.062	216	1.5	44
Astragaloside IV	807.159→627.419	216	1.5	46
Astragaloside IV	807.159→675.376	216	1.5	49

The HPLC-DAD-ELSD analysis of the Huangqi Granule samples were performed on an Agilent 1260 series instrument equipped with a quatpump, two detectors consisting of a DAD and an ELSD, and an auto-sampler. All of the data were collected and analyzed using ChemStation for LC 3D software. The chromatographic separations were performed on an Agilent Hypersil ODS column (4.6 × 250 mm, 5 *μ*m). The mobile phase consisted of water (A) and acetonitrile (B). The gradient program was as follows: 0–7 min, linear gradient 15–25% B; 7–12 min, linear gradient 25–32% B; 12–17 min, linear gradient 32–50% B; 17–30 min, isocratic 50% B; and 30–35 min, linear gradient 50–65% B. The wavelength of the UV detector was set at 254 nm with a flow rate of 1.0 ml/min. The volume injected was 50 *μ*L with a column temperature of 30°C.

## Results

### Antioxidant capacity assay

Table 2 displays the DPPH radical-scavenging activities of 30 batches of Huangqi Granules. The results show that the radical-scavenging activities of antioxidants were basically the same, with an IC_50_ rang of 2.317 mg to 2.860 mg and a mean IC_50_ value of 2.459 mg. This result indicates that different batches of Huangqi Granule have a stable antioxidant capacity.

**Table 2:** HPPH assay results.

Batch number	IC_50_ Values (mg)	Batch number	IC_50_ Values (mg)	Batch number	IC_50_ Values (mg)
NO_101102_	2.513	NO_110104_	2.366	NO_110402_	2.527
NO_101003_	2.382	NO_110105_	2.398	NO_110501_	2.415
NO_101101_	2.860	NO_110106_	2.319	NO_110502_	2.514
NO_111103_	2.432	NO_110201_	2.647	NO_110503_	2.447
NO_101103_	2.563	NO_110202_	2.475	NO_110504_	2.469
NO_101104_	2.445	NO_110203_	2.354	NO_110505_	2.558
NO_101201_	2.406	NO_110204_	2.430	NO_110601_	2.329
NO_110101_	2.363	NO_110301_	2.442	NO_110602_	2.317
NO_110102_	2.424	NO_110303_	2.422	NO_110603_	2.744
NO_110103_	2.433	NO_110401_	2.369	NO_110604_	2.405

### Improved immune ability analysis

The proliferation levels of RAW 264.7 cells in the presence of Huangqi Granules at concentrations of 31.3, 62.5, 125.0, 250.0, and 500.0 μg/mL for 24 h were 109.0 ± 1.3%, 115.8 ± 1.5%, 129.2 ± 2.1%, 130.9 ± 2.3%, and 142.2 ± 2.4%, respectively, (*p* < 0.001) of the normal group treated with medium only. These data suggest that Huangqi Granules might activate macrophages more safely than bacterial lipopolysaccharide (LPS) because LPS, which also increases intracellular calcium and H_2_O_2_ production in macrophages, also exerts cytotoxic effects on macrophages.

### Study of serum pharmacochemistry

**Fig 2:**
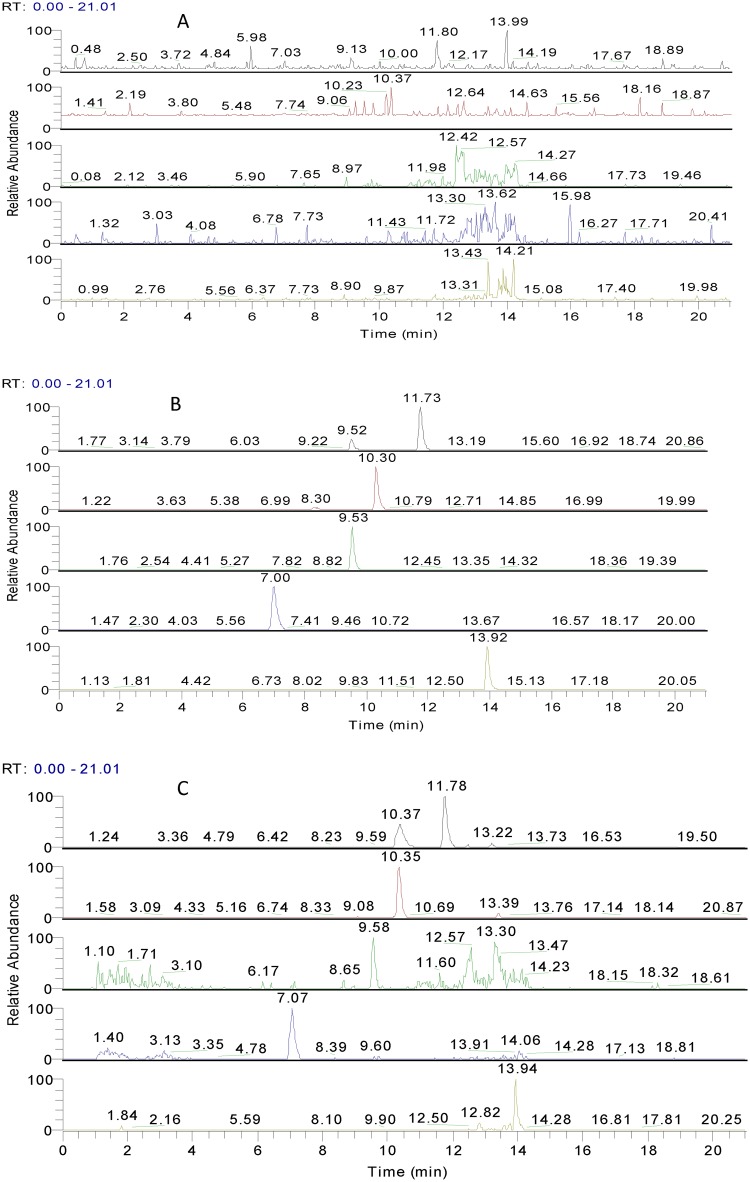
Serum pharmacochemistry analysis. A, rat serum before administration; B, blank serum + reference control; and C, 5 min after oral administration of Huangqi granule

The serum pharmacochemistry of the Huangqi Granule extract was first studied in the present work. As a result, calycosin-7-O-*β*-D- glucoside, ononin, calycosin, astragaloside IV, and formononetin were found to exist in rats’ blood 5 min, 15 min, and 30 min after dosing (Fig 2) and were designated biomarkers for the quality control of Huangqi Granules.

### Optimization of extraction

**Fig 3:**
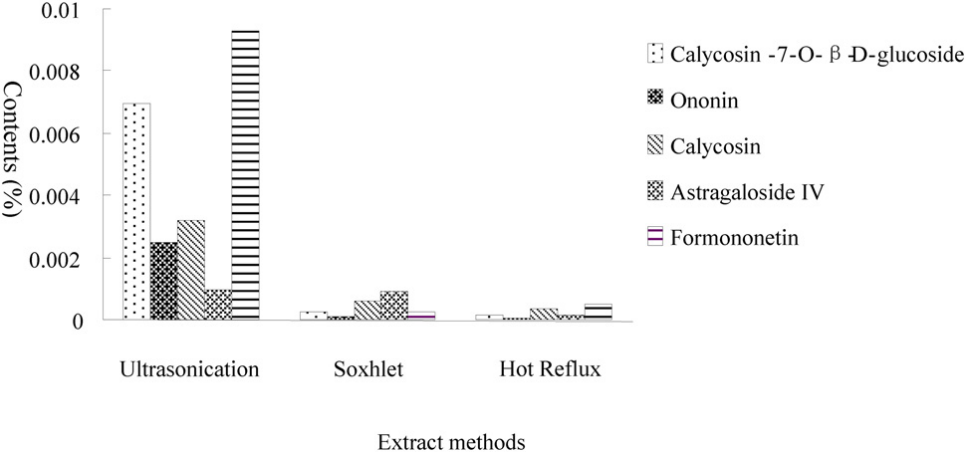
Comparison of the three extraction methods.

Three different methods, including ultrasonication, Soxhlet extraction and hot reflux extraction, were compared using the amount of the five chemical compounds as the indicator. The results shown in Fig 3 indicate that the best extraction effect was obtained using ultrasonication. Therefore, ultrasonication with 30% methanol as the extraction solvent was used.

### Optimization of the chromatographic conditions

**Fig 4:**
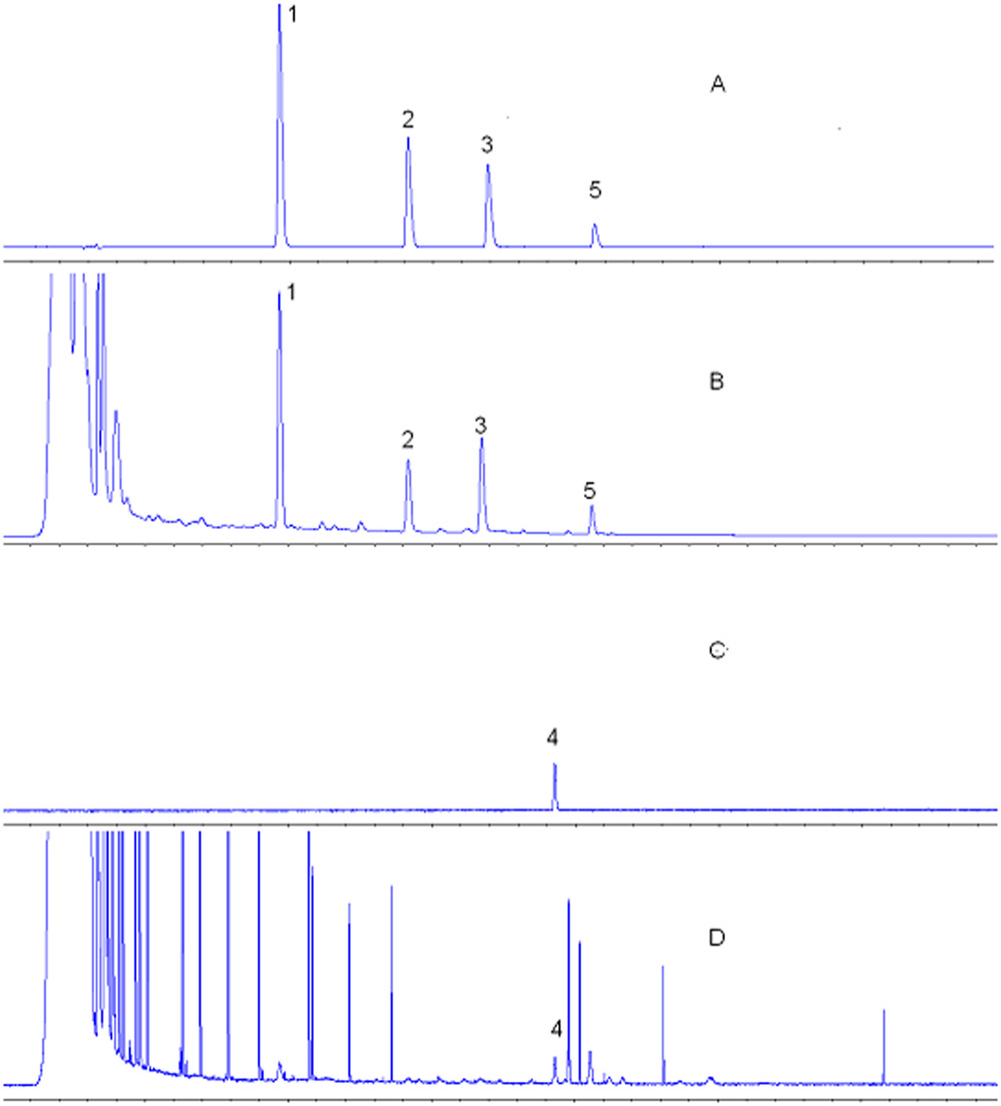
Representative HPLC chromatograms of the mixed standards and the Huangqi Granule extract detected using DAD and ELSD. (A) Mixed standards containing the four (nos. 1, 2, 3, and 5) chemical constituents in DAD; (B) Huangqi Granule extract at 254 nm in DAD. (Sample no. 110401); (C) standard of compound no. 4 in ELSD; (D) extract of Huangqi Granule in ELSD. (Sample no. 110401). Peaks: (1) Calycosin-7-O-*β*-D-glucoside; (2) Ononin; (3) Calycosin; (4) Astragaloside IV; and (5) Formononetin.

Four different types of columns, including an Agilent Hypersil ODS column (4.6 × 250 mm, 5 *μ*m), an Agilent Eclipse XDB-C18 column (4.6 × 150 mm, 5 *μ*m), a Phenomenex Synergi Fusion-RP column (4.6 × 250 mm, 4 *μ*m), and a Diamonsil C18 column (4.6 × 250 mm, 5 *μ*m), were compared using chromatographic separation as the main parameter. The results showed that the shortest retention time and the best peak type were obtained using the Agilent Hypersil ODS column (4.6 × 250 mm, 5 *μ*m). Different mobile phases, including methanol-water, acetonitrile-water, acetonitrile-water (0.5% formic acid), and acetonitrile-water (0.2% acetic acid), were tested; the acetonitrile-water system with gradient elution showed the best separation ability. Other parameters, such as the UV detection wavelength, drift tube temperature, and nitrogen pressure, were optimized in the present study. The optimized parameters were ultimately selected as shown in “instrumentation and chromatographic conditions”. Representative HPLC chromatograms are shown in Fig 4.

### Method validation

The working solutions were brought to room temperature and mixed using an autosampler before injection into the HPLC for the construction of calibration curves. Compounds 1, 2, 3 and 5 were detected using the DAD. The calibration curves were constructed by plotting the peak areas (*X*) versus the contents (*Y*) of each analyte. Meanwhile, compound 4 was detected using the ELSD. The calibration curve was constructed by plotting the Napierian logarithm of the peak area (*LN(X)*) versus the Napierian logarithm of the content (*LN(Y)*) of each analyte, according to the regression equation described by *LN(Y) = aLN(X) + b*. As shown in Table 3, the correlation coefficient (*R* ^*2*^) was ≥ 0.9972 for each calibration curve.

**Table 3:** Calibration curves.

Analyte	Calibration curves	*r*	Liner range(*μ*g)
Calycosin-7-O-*β*-D-glucoside	Y = 3831.4X+39.695	0.9999	0.1124~2.248
Ononin	Y = 4.58.4X-10.772	0.9996	0.0508~1.016
Calycosin	Y = 4981.9X-9.3962	0.9999	0.0357~0.714
Astragaloside IV	Y = 1.5918X+5.2847	0.9972	0.2242~4.484
Formononetin	Y = 4998.4X-0.4745	0.9993	0.00548~0.1096

Y is the peak area in DAD or logarithm of peak area in ELSD, and X is the concentration of the injected compound or the logarithm of the concentration of the injected compound.

Intra-day variations for six replicates within one day and inter-day variations over three consecutive days were used to determine the precision of the developed method. The *RSDs* of the peak areas were used to determine the precision. As shown in Table 4, the overall intra-day and inter-day variations were less than 2.37%, indicating that the precision was satisfactory.

**Table 4:** Precision, repeatability, stability, and recovery of the HPLC method for the determination of the five markers.

Analyte	Precision R.S.D (%) (n = 6)		Repeatability R.S.D (%) (n = 6)		Stability R.S.D (%)(n = 6)	Recovery (%) (n = 3)	
	intra-day(n = 6)	inter-day(n = 6)	Mean	R.S.D (%)		Mean	R.S.D (%)
Calycosin-7-O-*β*-D-glucoside	0.94	1.21	0.00713	2.24	0.61	98.56	0.65
Ononin	0.70	0.95	0.00250	1.30	0.80	99.49	2.07
Calycosin	1.39	1.79	0.0314	2.04	0.70	96.31	1.21
Astragaloside IV	2.37	2.11	0.00932	0.14	0.37	101.22	0.95
Formononetin	1.62	1.54	0.00097	2.78	1.71	100.32	1.66

To test the repeatability, six independent analytical sample solutions were prepared from sample no. 110401. Each sample solution was injected 3 times. All variations are expressed using RSD*s* as shown in Table 4, suggesting the method had good repeatability.

The same sample solution was injected into the apparatus every 2 h over 24 h to evaluate the stability. The *RSD* values of the peak areas of each compound were calculated and are shown in Table 4. All variations in the *RSDs* were less than 1.71%, indicating that the preparation of the samples solutions was relative stable at ambient temperature for 24 h.

Accuracy: A recovery test was used to evaluate the accuracy of the method. Six replications were performed on the five reference standards added into sample no. 110401 at the same concentration, which was labeled “Sample solution preparation”. The contents of the five reference compounds were measured individually by comparing the mean peak areas of the six samples with those of the extracted blank spiked with standards at corresponding concentrations. All analyses are expressed as *RSD* in Table 4, indicating that the method was accurate.

$$\text{Recovery}\;\left(\%\right)\;=\;(\text{amount}{\text{s}}_{\text{determined}}-\text{amount}{\text{s}}_{\text{original}})/\text{amount}{\text{s}}_{\text{spiked}}*100\%$$

Thirty batches of samples were determined using the established quantitative analytical method, and the contents were calculated and are summarized in Table 5. The results showed that the contents of each compound varied in different batches of Huangqi Granules. The contents of calycosin-7-O-*β*-D-glucoside, ononin, calycosin, astragaloside IV and formononetin ranged from 0.00258 to 0.00956%, 0.00173 to 0.00428%, 0.00046 to 0.01287%, 0.00376 to 0.01593% and 0.00004 to 0.00861%, respectively.

**Table 5:** The contents (% ± *S*.*D*.) of the five bioactive chemical compounds in 30 batches of Huangqi Granules samples.

Sample No.	Sample Batch No.	Calycosin-7-O-β-D-glucoside	Ononin	Calycosin	Astragaloside IV	Formononetin
1	110401	0.00707±0.00014	0.00248±0.00011	0.00315±0.00019	0.00934±0.00031	0.00098±0.00009
2	110202	0.00662±0.00036	0.00247±0.00030	0.00310±0.00026	0.01028±0.00037	0.00090±0.00003
3	110402	0.00956±0.00006	0.00428±0.00001	0.00169±0.00003	0.00951±0.00037	0.00057±0.00000
4	110502	0.00432±0.00006	0.00362±0.00003	0.00244±0.00005	0.00697±0.00015	0.00098±0.00000
5	110203	0.00807±0.00014	0.00285±0.00003	0.00202±0.00002	0.01593±0.00191	0.00147±0.00001
6	110103	0.00703±0.00011	0.00278±0.00004	0.00669±0.00001	0.01286±0.00180	0.00468±0.00000
7	110601	0.00323±0.00003	0.00276±0.00004	0.00290±0.00001	0.00709±0.00011	0.00243±0.00000
8	110603	0.00708±0.00016	0.00249±0.00002	0.00351±0.00001	0.01098±0.00055	0.00106±0.00004
9	101103	0.00761±0.00008	0.00269±0.00001	0.00181±0.00009	0.00953±0.00018	0.00048±0.00002
10	101002	0.00895±0.00016	0.00316±0.00003	0.00161±0.00002	0.01174±0.00057	0.00046±0.00002
11	110504	0.00315±0.00010	0.00241±0.00001	0.00048±0.00006	0.00461±0.00101	0.00006±0.00001
12	101201	0.00702±0.00016	0.00268±0.00001	0.00120±0.00000	0.01188±0.00017	0.00019±0.00000
13	110606	0.00350±0.00002	0.00282±0.00001	0.00064±0.00001	0.00648±0.00191	0.00059±0.00001
14	110104	0.00618±0.00010	0.00222±0.00010	0.00561±0.00018	0.01108±0.00028	0.00193±0.00004
15	110303	0.00843±0.00001	0.00294±0.00001	0.00506±0.00003	0.01032±0.00003	0.00174±0.00001
16	110501	0.00403±0.00005	0.00307±0.00001	0.00101±0.00001	0.00716±0.00031	0.00084±0.00001
17	110503	0.00347±0.00004	0.00284±0.00001	0.00113±0.00000	0.00739±0.00034	0.00072±0.00000
18	110604	0.00258±0.00002	0.00214±0.00001	0.00945±0.00013	0.00376±0.00116	0.00611±0.00002
19	110201	0.00614±0.00003	0.00202±0.00001	0.00385±0.00003	0.01003±0.00008	0.00120±0.00000
20	110102	0.00787±0.00007	0.00310±0.00002	0.00851±0.00002	0.01110±0.00012	0.00861±0.00000
21	110106	0.00469±0.00003	0.00175±0.00001	0.01287±0.00003	0.00730±0.00024	0.00690±0.00001
22	110204	0.00781±0.00016	0.00279±0.00001	0.00095±0.00005	0.01082±0.00037	0.00012±0.00000
23	110105	0.00623±0.00004	0.00222±0.00002	0.00188±0.00000	0.01101±0.00020	0.00044±0.00000
24	110101	0.00775±0.00003	0.00269±0.00001	0.00204±0.00001	0.01072±0.00019	0.00049±0.00002
25	101101	0.00679±0.00008	0.00246±0.00007	0.00046±0.00005	0.01071±0.00008	0.00004±0.00000
26	101102	0.00613±0.00002	0.00231±0.00007	0.00220±0.00002	0.01047±0.00015	0.00062±0.00000
27	110301	0.00616±0.00003	0.00238±0.00001	0.00836±0.00002	0.01043±0.00008	0.00572±0.00001
28	101003	0.00459±0.00001	0.00173±0.00002	0.00317±0.00003	0.00838±0.00007	0.00096±0.00001
29	110505	0.00266±0.00001	0.00222±0.00000	0.00028±0.00000	0.00565±0.00005	0.00010±0.00000
30	100401	0.00734±0.00002	0.00258±0.00001	0.00526±0.00000	0.00769±0.00013	0.00146±0.00001

### Statistical analysis

**Fig 5:**
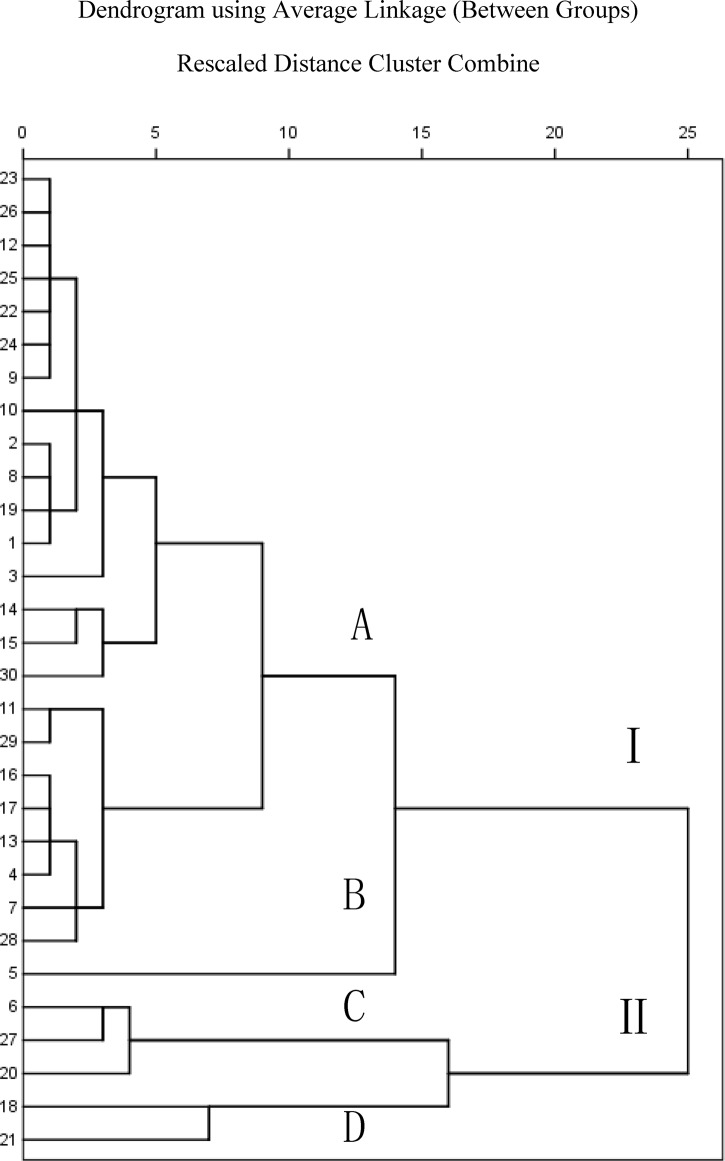
HCA dendrogram for the 30 Huangqi Granule batches.

Hierarchical clustering analysis (HCA) is a mathematical method that is used for case (different samples in our research) or variable (the contents of the five bioactive compounds) classification, and the results are expressed using a dendrogram [26, 27]. With the purposes of classification, HCA could be divided into two analyses: one is a class with samples, which is known as Q-type analysis, and the other is a class with variables, which is known as R-type analysis. R-type analysis was used in the present study. Thirty batches of samples were analyzed using SPSS (ver. 18.0), and the dendrogram is shown in Fig 5, indicating that all of the samples were divided into two clusters (Cluster I and Cluster II) with a correlation of 25.

Cluster I was divided into two subgroups (A and B). Subgroup B was composed of sample no. 110203, and subgroup A consisted of the other samples in this cluster. Cluster II was divided into subgroup C and subgroup D with a correlation of 15. Sample Nos. 1110103, 110301, and 110102 were in subgroup C, and samples Nos. 110604 and 110106 were in subgroup D. Cluster I, which consists of the other samples, was divided into subgroup A and subgroup B. Sample no. 110203 was the only class in subgroup B, and the other samples were in subgroup A. The contents of calycosin and formononetin in the samples from cluster I were greater than 0.00669% and 0.0047%, respectively. The contents of calycosin and formononetin in the samples in cluster II were lower than 0.00561% and 0.0024%, respectively. The contents of astragaloside IV in sample no. 110203 (subgroup B) was the highest out of all of the samples. In cluster II, the calycosin contents were greater than 0.0945% in subgroup D but lower than 0.00851% in subgroup C. Perhaps there were reasons for these classifications. For Cluster I and Cluster II, the most likely reason is that the main raw material of Huangqi granule has two legitimate sources [*Astragalus membranaceus* (Fisch.) Bge, var, mongholicus (Bge.) Hsiao and *Astragalus membranaceus* (Fisch.) Bge] in the current edition of Chinese pharmacopoeia. Meanwhile, subgroups were mostly caused by the quality differences of astragalus membranaceus raw material, which affected by the weather, geographical features, harvest time, etc.

## Discussions

Quality control research is one of the hot issues in the field of traditional Chinese medicine. At present, quality control for Chinese traditional medicine is given priority to monitoring the indexity components. However, as researchers in the field of TCM quality control, we should determine if the chemical compounds that were selected for quality control of an herb are exactly the ones absorbed into the patients’ blood and responsible for the pharmacological effects. Therefore, a serum pharmacochemistry study on Huangqi Granule was performed to screen for the absorbed constituents in the present study. Meanwhile, antioxidant capacity and immunocompetence of Huangqi Granule were also been researched. Based on the above research achievement, an HPLC method coupled with DAD and ELSD was developed for the simultaneous quantification of five bioactive chemical compounds in Huangqi Granules. The established method was efficient, simple and rapid, and it was used for the quantification of the five bioactive chemical compounds in the different samples. Combined with statistical analysis, a classification of the samples was performed, which was primarily explained by analyzing the original data.
